# Faster, more accurate? A feasibility study on replacing human judges with artificial intelligence in video review for the Paris Olympics Taekwondo competition

**DOI:** 10.3389/fspor.2025.1632326

**Published:** 2025-08-18

**Authors:** Yuncheng Zhang, Ruojie Qu, Olivier Girard

**Affiliations:** ^1^ School of Physical Education, Yan'an University, Yan'an, China; ^2^ Faculty of Business, Lingnan University, Tuen Mun, Hong Kong SAR, China; ^3^ School of Human Sciences (Exercise and Sports Science), The University of Western Australia, Perth, WA, Australia

**Keywords:** Taekwondo, artificial intelligence, Paris Olympics, sports referee, sports fairness

## Abstract

**Introduction:**

This study explores the potential of artificial intelligence (AI) to enhance the accuracy and efficiency of video review systems in Taekwondo, addressing limitations in current human-based judgment processes during competitions.

**Methods:**

A total of 241 video review cases from the 2024 Paris Olympic Taekwondo competition were analyzed. AI-based judgments were generated using ChatGPT-4.5 and OpenPose deep learning models. The AI-generated penalty decisions were statistically compared to those made by international video review referees using Cohen's Kappa coefficient.

**Results:**

The AI system demonstrated strong agreement with international referees (*κ* = 0.897, *p* < 0.001). Discrepancies occurred in only 9 out of 241 cases, primarily in scenarios involving head strikes with minimal contact or visual occlusion. Additionally, the AI system reduced average review time by approximately 81% by automatically identifying critical frames.

**Discussion:**

While AI significantly improved efficiency and showed high consistency with expert judgments, human oversight remains crucial for ambiguous or complex cases. A hybrid model—AI-assisted pre-review followed by referee confirmation—is proposed to optimize decision-making. Future developments should focus on real-time detection, multi-angle video integration, and application to other sports such as baseball, basketball, boxing, and judo.

## 1 Introduction

As a globally recognized Olympic combat sport, Taekwondo ranks among the most popular martial arts. Taekwondo debuted at the 1988 Seoul Olympics as a demonstration sport and became an official Olympic event in 2000 (1). Since then, the competition rules have been updated more than ten times to align with Olympic standards and enhance fairness and transparency (2, 3). A major technological advancement came in 2007 when World Taekwondo introduced the Protective Scoring System (PSS) and Video Replay System (VRS), significantly advancing the overall development of Taekwondo (4, 5).

The PSS uses electronic sensors to objectively record trunk strikes. When sufficient force is applied through contact between an athlete's electronic foot protectors and the opponent's sensor-equipped body gear, points are automatically awarded (6). This reduces scoring errors from subjective judgments, mitigates referee shortages, and improves scoring reliability (3, 4, 7). The VRS enables coaches to challenge decisions such as “*a successful head strike not detected by the electronic headgear”*, “*my athlete not committing a foul”*, or “*the opponent committing a foul”.* A replay judge reviews the footage to determine the validity of each challenge. By encouraging the use of head-scoring techniques, the system increases spectator appeal and helps reduce referee error (8, 9). However, inconsistencies in rule interpretation and judging criteria mean VRS decisions can still be influenced by subjectivity, limiting their ability to ensure full impartiality and objectivity.

Subjective referee decisions impact competitive fairness not only in Taekwondo but across many sports. Despite regulations aimed at reducing subjectivity, human error in officiating remains unavoidable (9–11). For instance, Krustrup and Bangsbo (2001) found that soccer referees call fewer fouls in the second half of matches. They attributed this decline to physical fatigue from the intense running demands of the first half, which may reduce focus and increase the likelihood of missed calls (12, 13).

To address subjective officiating challenges in Taekwondo, sports administrators are increasingly adopting advanced technologies that delegate some decision-making to intelligent computer systems (14). These systems use high-definition cameras to monitor competition, detect fouls, and conduct frame-by-frame analysis, delivering rulings based on predetermined rules (15–17). With their computational power and reliability, artificial intelligence (AI) systems could diminish inconsistencies in human officiating. By applying consistent, predefined criteria, AI could minimize potential bias and enhance fairness in officiating decisions (9, 10, 13). This phenomenon was also revealed by Wang & Hsieh (2016). They found that in NBA games, the accuracy of referees' decisions was significantly affected by the stage of the game (the error rate increased in the last two minutes) and the home field effect (18).

AI technology is increasingly adopted in sports such as soccer, basketball, fencing, and baseball, where it has received positive evaluations (9, 18). One notable example is the fencing adjudication system, where AI achieves 90% accuracy after just three hours of training, significantly reducing the time required from human referees (19). In soccer, the VAR system has been integrated into major competitions such as the World Cup, UEFA Champions League, and La Liga to help identify offside, handball, and out-of-bounds infractions. By offering instant review footage and 3D reconstruction, VAR likely enhances transparency and fairness, with many spectators expressing greater trust in AI decisions over human refereeing (20, 21).

This study leverages recent technological advancements and the unique opportunity of the Paris Olympics to reassess 251 video reviews from the Taekwondo competition using AI. By comparing AI-generated decisions with those of international-level video review judges, we evaluated the stability and accuracy of AI in Taekwondo officiating. Although AI judgment has been initially explored in sports such as football and baseball, it is the first time in combat sports, especially in taekwondo competitions. In addition, this study used the most advanced algorithm modes of ChatGPT-4.5 and OpenPose to ensure the reliability of the experiment. Based on existing literature, two hypotheses were formulated: H1.The AI video review system will maintain an error rate of 2% in penalty decisions; H2.AI will help reduce Taekwondo video review process by more than 50% (22, 23). Our intention was also to explore AI's potential applications in the video review process and broader refereeing practices. Finally, this research identifies current shortcomings in AI decision-making, analyzes their causes, and proposes solutions, thereby providing a theoretical framework for future developments in this field.

## 2 Methods

### 2.1 Study design

This study analyzed 251 video reviews from the 2024 Paris Olympics Taekwondo event. Of the 255 initial reviews, four were simultaneously challenged by both coaches, resulting in 251 unique cases. Due to broadcast issues, 10 videos were unusable, leaving 241 clips (ranging from 3 to 19 s) for the final analysis.

All videos were sourced from the original broadcast to match the content and viewing angles seen by video review judges. During the Paris Olympics, the judges shared their computer screens through split-screen broadcasts to ensure transparency, and videos for this study were sourced from these shared screens. After recording whether each ruling was successful, segments showing the judges' final decisions (i.e., including hand gestures indicating outcomes) were removed from the 241 videos to prevent visual cues from influencing the AI's judgment.

### 2.2 Experimental tools

This study utilized ChatGPT-4.5 with Canvas as the primary experimental tool. Released in February 2025, ChatGPT-4.5 is the latest AI analysis model from ChatGPT. The experiment incorporated pixel overlap analysis using the HSV color space and key point detection techniques to locate the athlete's foot and the opponent's helmet. Key frames with a foot-to-helmet distance of 10 pixels or less were analyzed, along with dynamic motion analysis to detect potential contact moments.

The OpenPose model was also employed for in-depth analysis. It leverages convolutional neural networks (CNN) to extract features from input images, generating body key point confidence maps and Part Affinity Fields (PAFs). Confidence maps estimate the likelihood of specific points (e.g., nose, eyes, and shoulders), while PAFs describe the spatial relationships between them. The main algorithm is outlined below:

1. Image Input: An image or video frame *I* is provided as input, and the CNN extracts the feature map *F(I)*.
2. Generation of Confidence Maps and PAFs: Convolution operations generate key point confidence maps C*_k_*and Part Affinity Fields (PAFs) P*_i,j_*. Here, C*_k_* represents the probability of the presence of the *k*-the keypoint, while P*_i,j_* indicates the strength of the connection between two key points:
  $$C_{k}=f(F(I))\;P_{i,j}=g(F(I))$$

Here, *f* and *g* are trained convolutional operations used to generate the confidence maps and PAFs, respectively.

3. Loss Functions: To optimize the network, OpenPose uses two loss functions:

Keypoint Confidence Map Loss *L*_conf_: Measures the error between the predicted key points and the actual key points.

$$L_{\text{conf}}=\overset{k}{\underset{k=1}{\sum }}\;||C_{k}^{\mathrm{pred}}-C_{k}^{\mathrm{gt}}|{|}^{2}$$

PAF Loss *L*_PAF_: Measures the accuracy of the predicted connections between key points.

$$L_{\text{PAF}}=\underset{i,j}{\sum }\;||P_{i,j}^{\mathrm{pred}}-P_{i,j}^{\mathrm{gt}}|{|}^{2}$$

The total loss function is defined as:

$$L=\alpha L_{\mathrm{conf}}+\beta L_{\mathrm{PAF}}$$

Where *α* and *β* are hyperparameters used to balance the two loss components.

4. Multi-Stage Optimization: OpenPose employs a multi-stage iterative approach for optimization. At each stage, the network refines its predictions based on the results from the previous stage, gradually improving the accuracy of key point locations and PAFs to ensure precise pose estimation.
  $$\mathrm{Iterative}\;\mathrm{Refinement}:\;C_{k}^{\mathrm{new}}=f(C_{k}^{\mathrm{old}},P_{i,j}^{\mathrm{old}})$$
5. Skeleton Assembly: Using a greedy algorithm, the connections between key points are determined based on PAFs to assemble a complete skeleton structure. The algorithm selects the optimal connection paths according to PAF strength, linking key points together to generate the human pose diagram.

### 2.3 Experimental steps

**Figure 1:**
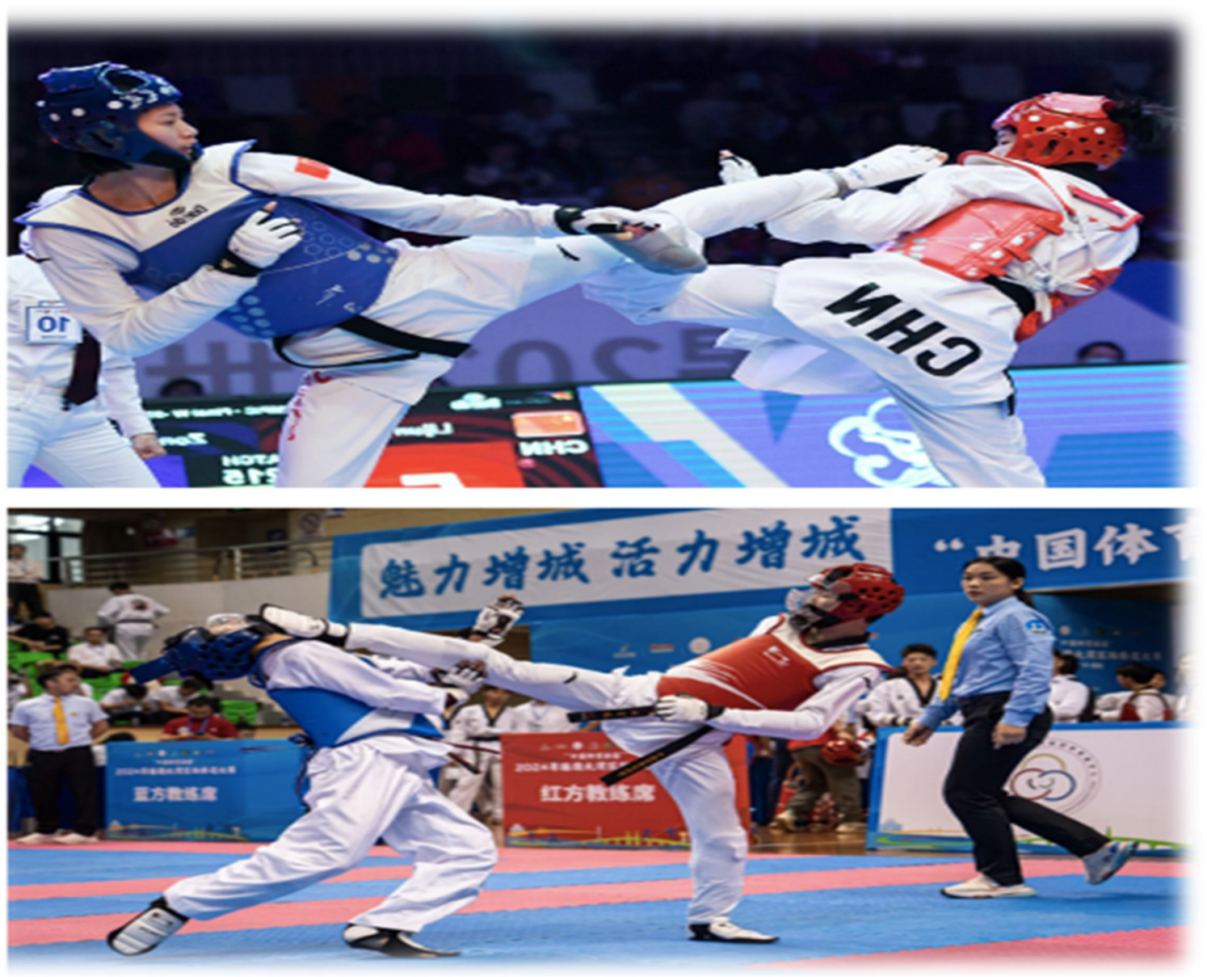
Ai deep learning test pictures: the top picture shows a failed head strike, while the bottom picture depicts a successful head strike (reproduced with permission from the International Olympic Committee, Women's −49kg vs Men's −58kg Repechage/Final | Taekwondo | Paris 2024 Olympics).

AI Deep Learning Phase: Before the experiment, the AI was given the latest version of the Taekwondo competition rules and instructed to thoroughly learn all the rules, with a particular focus on video review regulations. It then processed 300 randomly presented high-resolution images (∼200 KB each) from past Taekwondo competitions. For each image, the AI determined whether the athlete executed a successful head strike, stepped out of bounds, or fell. The dataset included: 100 successful head strikes, 100 failed head strikes, 25 out-of-bounds, 25 in-bounds, 25 falls, and 25 no falls. This dataset of 300 images was selected from Olympic-level competitions, covering all weight classes and genders, and included photos of youth competitions to ensure that the AI can receive the most comprehensive recognition training (Figure 1). After each judgment, the AI received feedback – correct decisions were reinforced positively, while incorrect ones prompted the AI to explain its reasoning, enabling iterative learning and refinement of its decision-making process.

**Figure 2:**
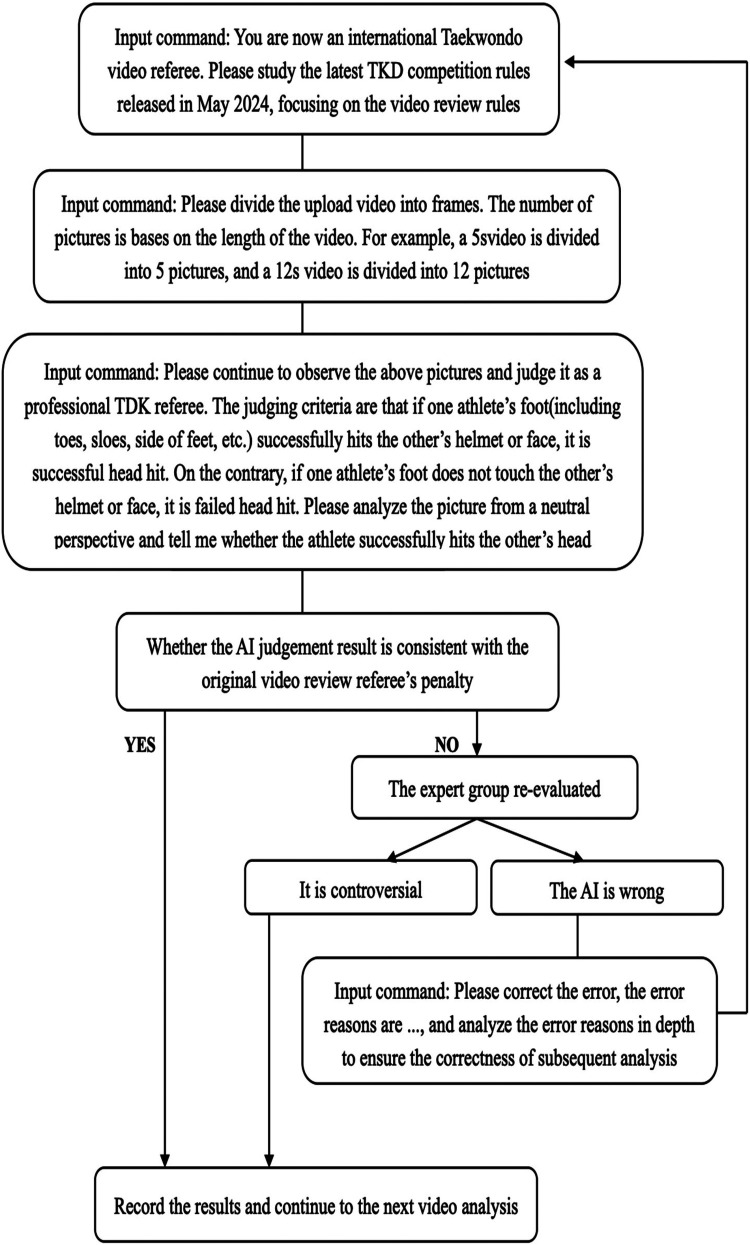
Pre-experiment operation flowchart.

Pre-Experiment Phase: After the AI learning phase, a pre-experiment was conducted to validate the set-up for the main experiment. It involved analyzing all video review recordings from the first day of the four-day 2024 Paris Olympics Taekwondo event. Of the 63 videos reviewed, the AI's judgments matched those of the on-site video review judges in 59 cases, resulting in a 93.65% agreement rate. The pre-experiment steps are illustrated in Figure 2.

During the pre-experiment, the AI's analysis differed from the judges' assessments in four cases – all involving head strikes that the judges ruled as successful, but the AI incorrectly classified as failures. An expert panel reviewed the discrepancies and confirmed them as “AI judgment errors”. Consequently, the AI was updated with a new the guideline: “*even if the athlete's toes make slight contact with the opponent's electronic headgear, it should be considered a successful head strike.”* After incorporating this rule, the AI re-evaluated the four videos, and its revised judgments aligned with those of the judges.

**Figure 3:**
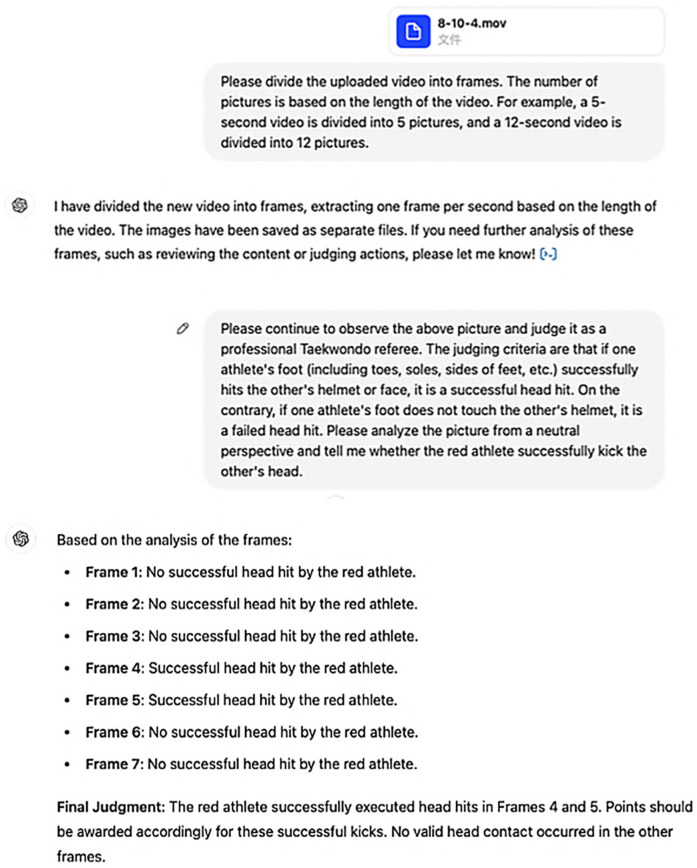
Part of the experimental pictures.

Experimental Phase: The formal experiment analyzed all video review recordings from the final three days of the 2024 Paris Olympics Taekwondo event, totaling 178 videos. One video was excluded due to file corruption, leaving 177 videos (*N* = 177) for final analysis. Unlike the pre-experiment, the formal phase did not include detailed re-evaluation of discrepancies between AI and on-site judges. Instead, all results were recorded immediately after the AI made its judgment. Figure 3 presents images from parts of the experiment.

Data Analysis: Following the experiment, penalty decisions were categorized into nine major types, including “head strike”, “out of bounds”, and “rotation technique score”. Cases where both the AI and international-level referees deemed the decision successful were marked as “Yes” and unsuccessful cases as “No”. The AI's decisions were then compared with those of on-site international referees, and the agreement rate−defined as the proportion of “Yes” or “No” verdicts−was calculated to evaluate decision accuracy. Statistical analyses were conducted using SPSS 27. Descriptive statistics were employed to calculate and analyze penalty outcomes and relevant indicators that could influence adjudication results, providing a comprehensive overview of data distribution and forming the basis for subsequent inferential analyses. Cohen's Kappa coefficient (*κ*) was employed to assess overall agreement between the AI and referees, with separate *κ* values calculated for each penalty category to assess consistency across sub-items. For interpretation, *κ* values were classified according to Landis & Koch (1977) as follows: *κ*<0 indicated *no agreement*; 0 ≤ *κ* ≤ 0.20, *slight*; 0.21 ≤ *κ* ≤ 0.40, *fair*; 0.41 ≤ *κ* ≤ 0.60, *moderate*; 0.61 ≤ *κ* ≤ 0.80, *substantial*; and 0.81 ≤ *κ* ≤ 1.00, *almost perfect* agreement. A Chi-square test was applied to determine the statistical significance of agreement rate differences, with *p* < 0.01 considered *highly significant*, *p* < 0.05 considered *significant*, and *p* ≥ 0.05 considered *non-significant*. After removing outliers and testing for normality, Pearson's correlation coefficient (*r*) was used for data meeting linearity and normality assumptions, while Spearman's rank correlation coefficient (*ρ*) was applied otherwise. Correlation strength was interpreted per Cohen (1988): |*r*| < 0.10, *very weak*; 0.10 ≤ |*r*| < 0.30, *weak*; 0.30 ≤ |*r*| < 0.50, *moderate*; 0.50 ≤ |*r*| < 0.70, *strong*; 0.70 ≤ |*r*| < 0.90, *very strong*; and 0.90 ≤ |*r*| ≤ 1.00, *nearly perfect*. Bland-Altman analysis was performed to evaluate systematic bias between the AI and referees by examining the mean difference and limits of agreement (mean difference ± 1.96 × SD). Agreement was considered acceptable if most differences fell within these limits and the mean was close to zero. Systematic discrepancies indicated potential bias. Finally, video cases with discordant AI-referee decisions were reviewed by an expert panel, consisting of one International-Class Taekwondo Competition Referee, one National-Class Taekwondo Competition Referee, one Senior Taekwondo Coach, and two Intermediate Taekwondo Coaches). This review aimed to identify causes and provide empirical evidence for optimizing the AI decision model, enhancing its accuracy and feasibility (24–26).

## 3 Results

**Figure 4:**
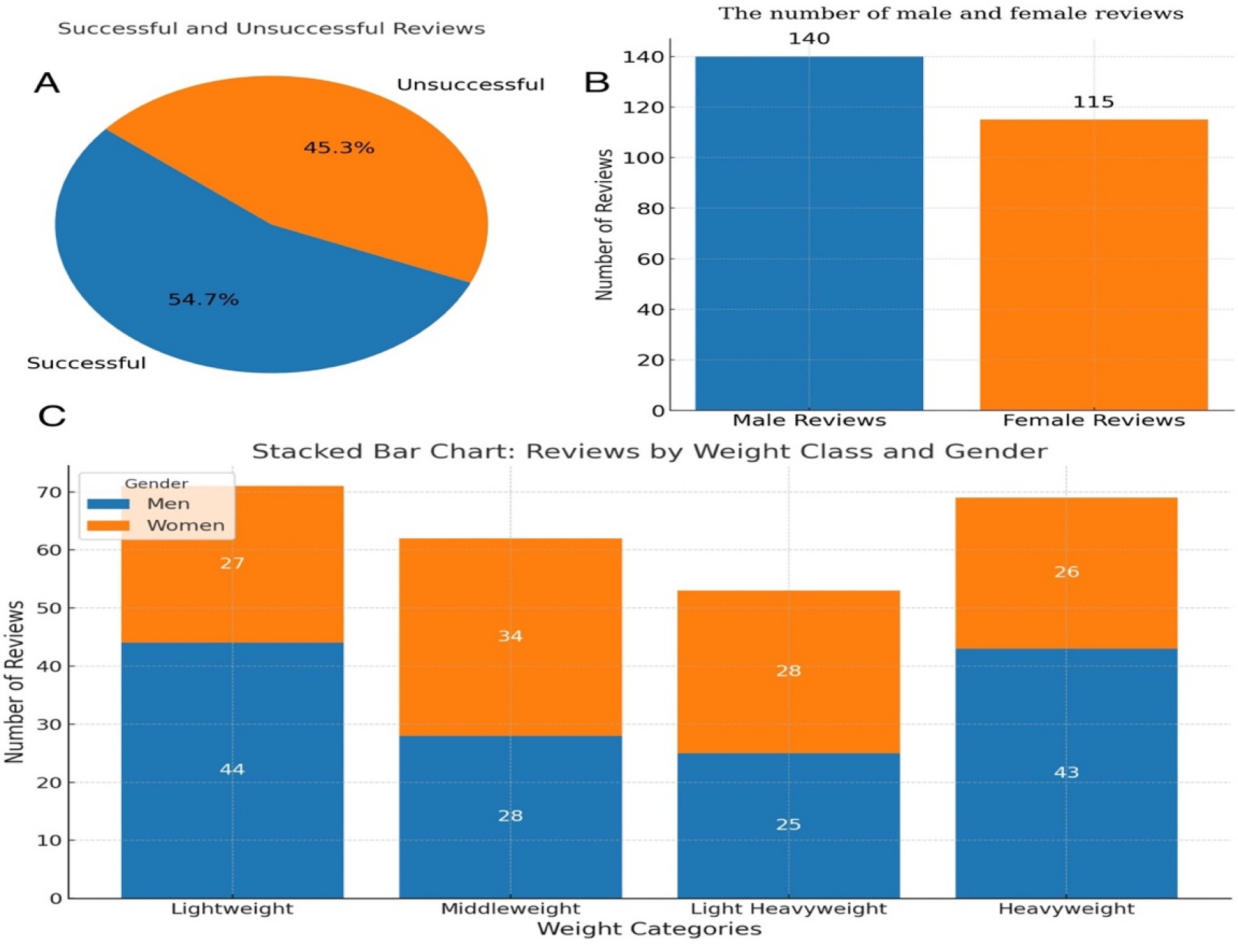
Video review data from the Paris Olympic taekwondo competition - percentage of successful and unsuccessful video reviews **(A)**; the number of reviews for male and female athletes **(B)**; and number of reviews by category **(C)**.

**Figure 5:**
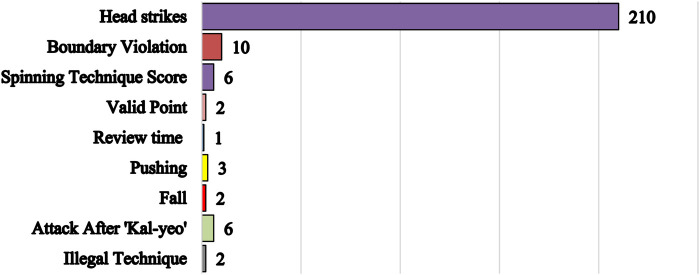
Categories of video reviews during the taekwondo competition at the 2024 Paris olympics.

During the 2024 Paris Olympics Taekwondo competitions, 255 video reviews were conducted, including cases where coaches from both sides simultaneously requested a review. As shown in Figure 4, 139 challenges were successful, while 115 were unsuccessful. Male athletes accounted for 140 instances, significantly more than the 115 for females. Among male weight categories, lightweight and heavyweight divisions had the highest review frequencies, representing 62% of all male challenges. For females, the middleweight and light heavyweight divisions accounted for 54% of challenges. Head contact reviews predominated, with 210 instances (82% of all reviews), as illustrated in Figure 5.

As shown in Table 1, the mean values of referee judgments and AI determinations were 0.55 and 0.56, respectively, indicating remarkable consistency between the two evaluation systems. The mean difference of −0.0169 suggests only minor deviations in the AI's judgments, with a negligible overall discrepancy. Both systems demonstrated nearly identical dispersion, with SD of 0.499 (referees) and 0.497 (AI), and corresponding population variances of 0.249 and 0.247. The SD (0.22549) and variance (0.051) of the differences (AI-referee) indicated moderate inter-case variability in scoring discrepancies, although the average deviation was minimal. Overall, the closely aligned central tendencies, near-identical dispersion, and near-zero mean difference demonstrate a high level of agreement between human and AI judgments.

**Table 1:** Descriptive statistics of video review referees and AI judgment results.

Variable	*N*	Sum of squares (SS)	Mean		Standard deviation (SD)	Population variance
			Statistics	SE		
Referees	177	97	.55	.038	.499	.249
AI	177	100	.56	.037	.497	.247
Difference	177	−3.00	-.0169	.01695	.22549	.051

An examination of the data in Table 2 reveals a *very strong*, statistically significant positive correlation between referee judgments and AI determinations, with a Pearson correlation coefficient (*r*) of 0.898 (*P* < 0.001 in two-tailed testing). The covariance between referees and AI reached 0.223, indicating high co-variation. Additionally, the Sum of Squares and Cross Products (SSCP) value of 39.198 further supports this *very strong* association. With a sample size of 177, these results show a statistically significant and *nearly perfect* linear relationship between referee judgments and AI determinations.

**Table 2:** Statistics on the correlation between video review referees and AI judgment results.

Variable	Statistic	Referees	AI
Referees	*P*-value	1	.898**
	Significance (2-tailed)		.000
	SSCP	43.842	39.198
	Covariance	.249	.223
	*N*	177	177
AI	*P*-value	.898**	1
	Significance (2-tailed)	.000	
	SSCP	39.198	43.503
	Covariance	.223	.247
	N	177	177

** At the 0.01 level (two-tailed), the correlation is significant.

**Figure 6:**
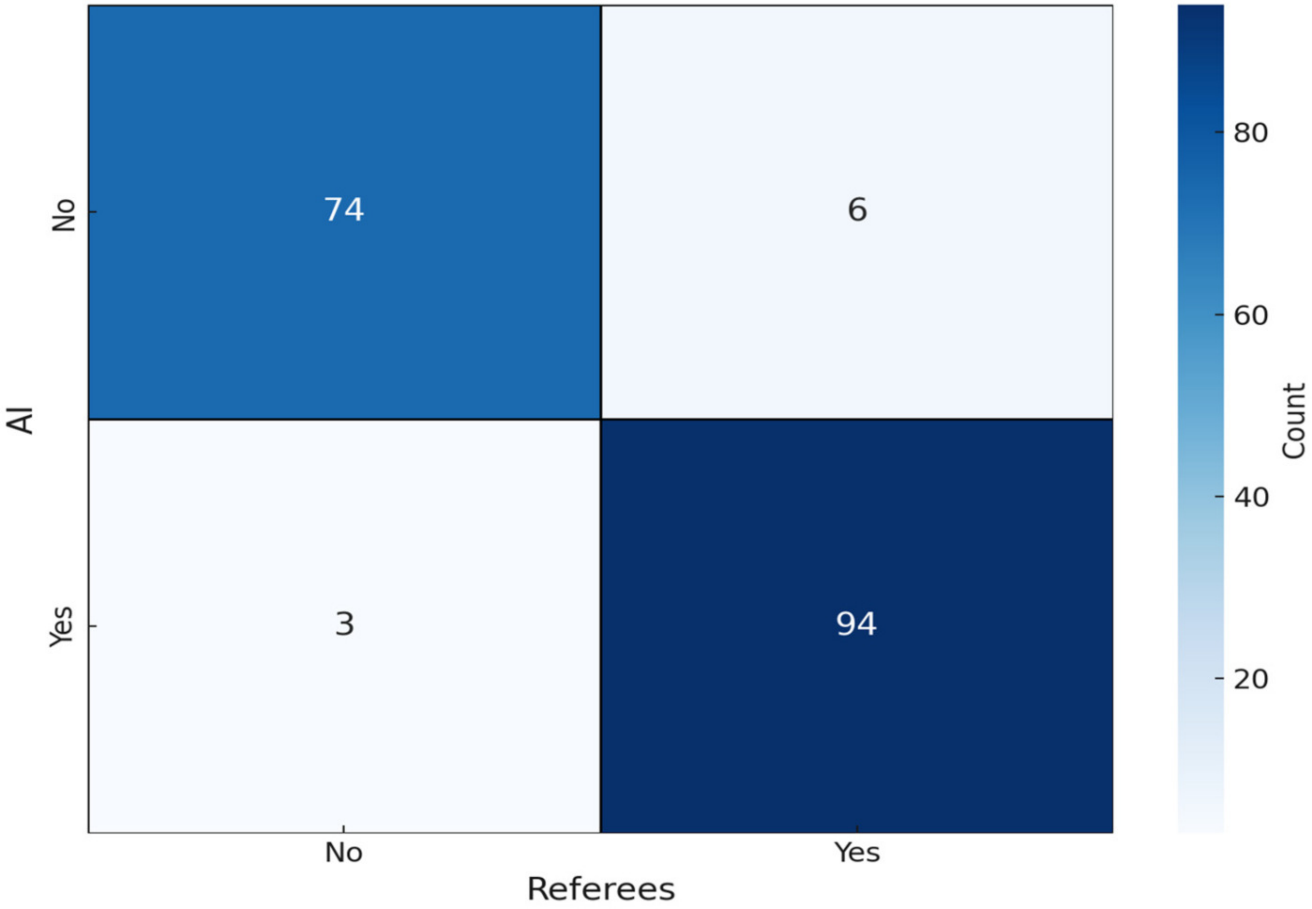
Heat map of AI and referee decisions.

The paired sample proportion test (Table 3) in this study showed a minimal proportion difference of −0.017 between referee and AI determinations, with a *Z*-score of −1.000 (one-tailed *P* = 0.159; two-tailed *P* = 0.317). Cross-tabulation in Table 4 and the heat map in Figure 6 demonstrate remarkable congruence in “No/Yes” judgments. When referees ruled “No”, the AI also judged “No” in 74 instances (compared to an expected 34.8 under the independence assumption). Only 6 cases showed a “No” from the referee and a “Yes” from AI, well below the expected 45.2. When referees decided “Yes”, the AI agreed 94 times (expected 54.8), with only 3 “Yes-No” discrepancies (expected 42.2). This concentration of observations along the diagonal (“No-No” and “Yes-Yes”) and sparse off-diagonal occurrences (“No-Yes” and “Yes-No”) demonstrates low bias and high consistency. Furthermore, marginal totals (e.g., 80 “No” by referees vs. 77 by AI) showed negligible variation, further reinforcing the high consistency between human and AI judgments.

**Table 3:** Paired sample proportion test.

Condition	Test type	Difference in proportions	Asymptotic standard error (ASE)	*Z*	Significance (*p*-value)	
					One-tailed *p*-value	Two-tailed *p*-value
Comparing referee and AI decisions	Mid-P adjusted *P*-value for Binomial test	−.017	.017		.172	.344
	McNemar's test	−.017	.017	−1.000	.159	.317

**Table 4:** Cross-tabulation of referee judgment and AI judgment.

Variable			AI		Count
			No	Yes	
Referees	No	Count	74	6	80
		Expected count	34.8	45.2	80.0
	Yes	Count	3	94	97
		Expected count	42.2	54.8	97.0
Count		Count	77	100	177
		Expected count	77.0	100.0	177.0

Based on 177 valid cases, the symmetry test between referee and AI judgments revealed a significant relationship, with a Kappa value of 0.897 (standard error = 0.033, *t* = 11.941, *p* < 0.001), demonstrating substantial agreement between the two systems (Table 5). The final chi-square test (Table 6) showed a Pearson chi-square value of 142.590, a continuity correction of 138.976, and a likelihood ratio of 172.993, with Fisher's exact test and linear association test yielding a value of 141.785, all resulting in *p* < 0.001. Referee and AI judgments were not independent in the 2 × 2 contingency table, as the Pearson Chi-Square statistic reached 142.590 (DF = 1) with a two-tailed significance of *p* < 0.001. Both the Continuity Correction and Likelihood Ratio tests displayed similarly high statistical significance, while Fisher's Exact Test and Linear-by-Linear Association tests all resulted in *P* < 0.001. These results indicate that, among the 177 valid cases, the distribution of referee and AI judgments significantly deviated from the “complete independence” hypothesis. This suggests a strong concordance between the two evaluation systems, rather than random or unrelated determinations.

**Table 5:** Symmetry test between referee judgment and AI judgment.

Test type	Value	Asymptotic standard error^a^	Approximate T^b^	Asymptotic significance
Measure of agreement - Kappa	.897	.033	11.941	.000
Valid cases	177			

^a^ The null hypothesis is not assumed.

^b^ The asymptotic standard errors are used with the null hypothesis assumed.

**Table 6:** Chi-square test of referee and AI judgment results.

Test type	Value	Degrees of freedom (DF)	Asymptotic significance (2-tailed)	Exact significance (2-tailed)	Exact significance (1-tailed)
Pearson Chi-Square	142.590^a^	1	<.001		
Continuity correction^b^	138.976	1	<.001		
Likelihood ratio	172.993	1	<.001		
Fisher's exact test				<.001	<.001
Linear-by-linear association	141.785	1	<.001		
Valid cases	177				

^a^ 0 cells (.0%) have an expected count less than 5. The minimum expected count is 34.80.

^b^ Calculation is only performed for 2 × 2 tables.

## 4 Discussion

Our main finding is that AI demonstrates decision-making capabilities nearly identical to those of international-level referees during Taekwondo video review processes. It showcases high stability and reliability, which proves its competency in handling video review tasks for Taekwondo competitions. In particular, AI's advantages in decision speed and objectivity are unattainable by human referees. In terms of speed, statistical data from the Paris Olympics show that a total of 255 video review instances occurred over the 4-day Taekwondo competition, which included 158 matches, averaging more than one video review per match. Each video review took between 47 s and several minutes, with the longest review exceeding 30 min. In comparison, a single round of a Taekwondo match (i.e., which follows a best-of-three system) lasts only 2 min. Therefore, excessive video reviews may significantly delay the overall progress of matches, prolong athletes' competition durations, and increase their physical and mental exhaustion. During video reviews, athletes must remain in the competition area while awaiting review results and resume the match immediately afterward without being allowed to drink water or sit down. This situation adversely affects their competition rhythm and state. The application of AI can address this issue. In experiments, AI analyzed each review video in just 3–9 s, with minor fluctuations due to network conditions, still far exceeding manual review speed. If AI was implemented in the video review process, it could be nearly four times faster than manual review, significantly reducing the pause caused by reviews and enhancing the overall fluency of the adjudication process.

AI technology significantly enhances the objectivity of video review decisions by strictly following predefined algorithms, eliminating subjective bias. This aligns with the design philosophy of electronic protectors, which remove human interference, and is consistent with the overall direction of Taekwondo competition. The Taekwondo judging system has evolved from relying on three corner judges making decisions based on visual and auditory cues (i.e., requiring at least two affirmative calls), which suffered from high error rates due to fast kicking speeds (up to 16 m/s) and visual obstructions (10, 27), to the current electronic protector system that ensures objectivity in torso scoring. While the current video review system aims to encourage high-difficulty head strikes (i.e., compensating for electronic head sensor limitations in detecting high-speed, small-contact-area impacts (22, 28), it still faces challenges. These include hardware limitations (i.e., international competitions use four 4K cameras, while most events only deploy two with inconsistent resolutions) and inconsistencies in refereeing standards – issues that persisted even during Paris Olympic head strike reviews. AI can overcome these limitations through pixel-overlap analysis, enabling reliable identification even with low-quality video or minimal contact area. Its algorithm-driven decision process guarantees objectivity on par with electronic protectors, addressing current subjectivity issues and providing reliable technical assurance for competition fairness.

**Figure 7:**
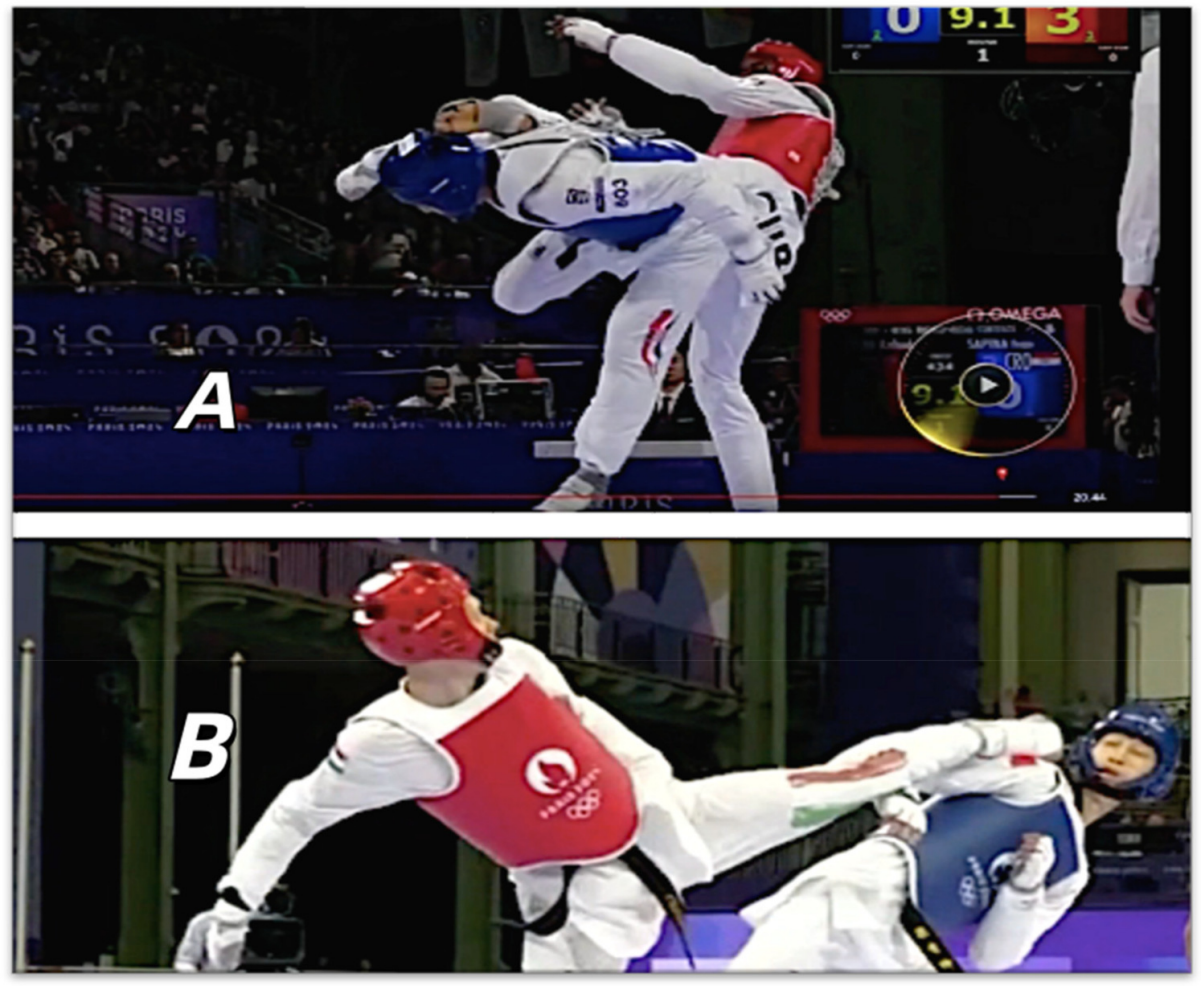
Ai judgment error diagram: AI incorrectly judged head strikes as failed **(A)**; AI correctly judged that head strikes as successful **(B)** (reproduced with permission from the International Olympic Committee, Women's −49kg vs Men's −58kg Repechage/Final | Taekwondo | Paris 2024 Olympics).

**Figure 8:**
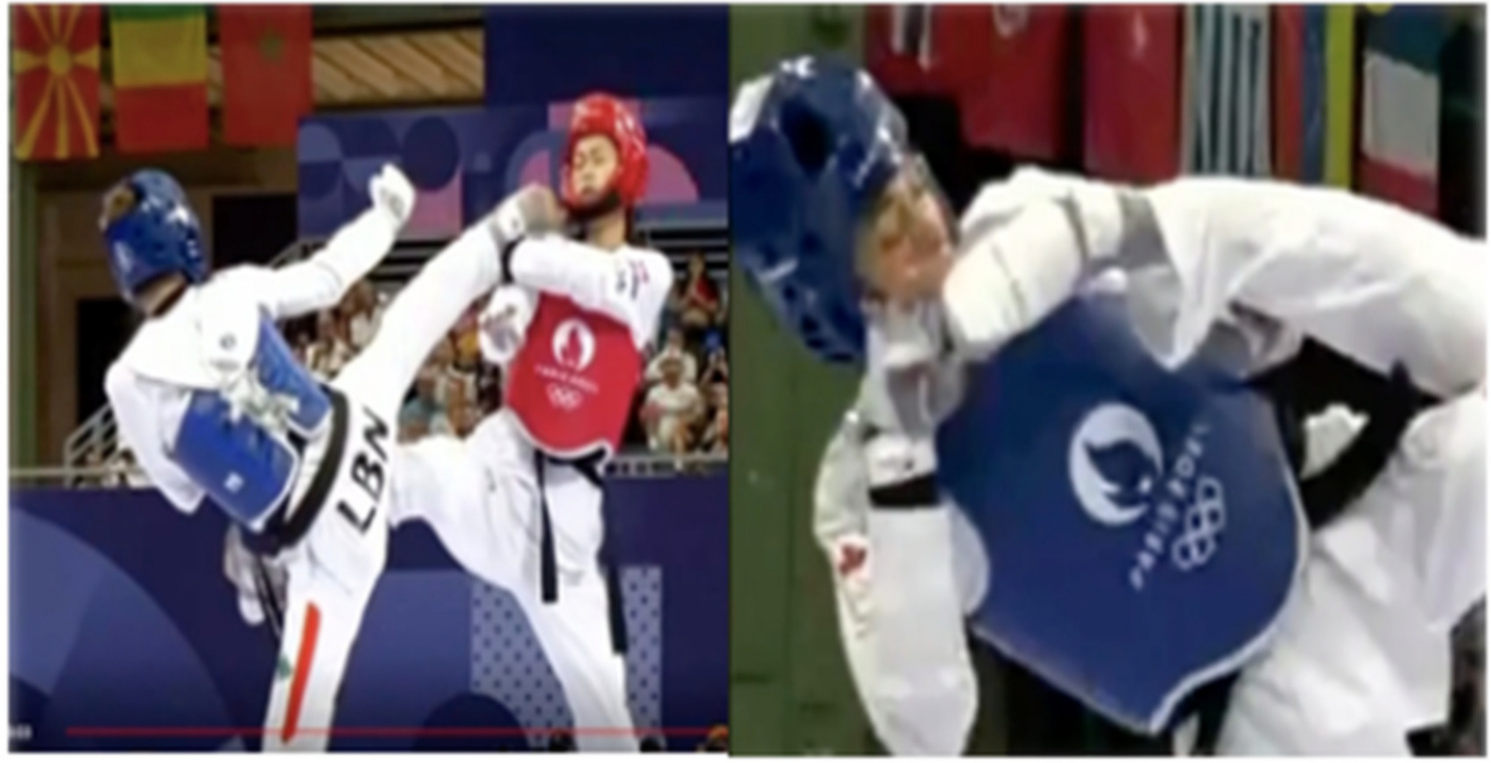
The video review: the referee judged the head strike as successful, while the AI judged it to be unsuccessful (reproduced with permission from the International Olympic Committee, Women's −49kg vs Men's −58kg Repechage/Final | Taekwondo | Paris 2024 Olympics).

**Figure 9:**
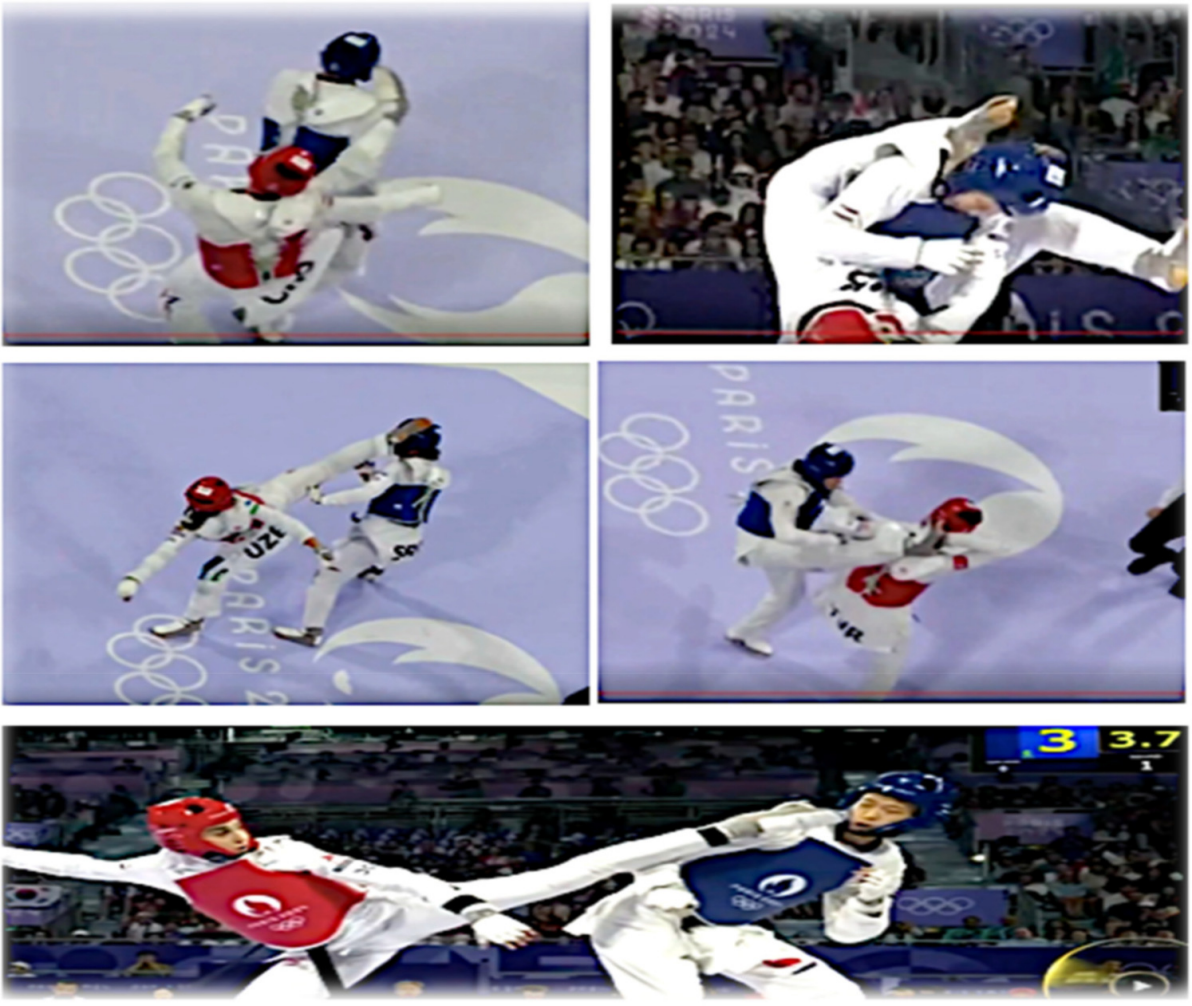
The video review: the referee determined that the head strike was unsuccessful, while the AI judged it as successful (reproduced with permission from the International Olympic Committee, Women's −49kg vs Men's −58kg Repechage/Final | Taekwondo | Paris 2024 Olympics).

Another observation was that AI adjudication, with its decision-making speed and objectivity, possesses inherent advantages in video review processes and aligns with the evolving needs of Taekwondo competitions. However, for AI to fully replace human judgment, its accuracy and stability must be ensured. The video review process is critical in Taekwondo, particularly because head strike reviews, accounting for 82% of all reviews, can instantly alter match outcomes, with a successful head strike review awarding 3 points to the reviewing party. Other reviewable actions similarly lead to immediate point adjustments (1–2 points), making absolute consistency in adjudication imperative for this decisive procedure (7). However, Figure 6 reveals nine (Women's 57 kg 3 times; Women's 67 kg 2 times; Men's + 80 kg 2 times; Men's 68 kg 1 time; Women's + 67 kg 1 time) persistent discrepancies between AI adjudication and international-level referees' decisions. A thorough analysis of these discrepancies is essential for evaluating the AI system's operational reliability in competitive settings and identifying specific areas for improvement. Following expert panel discussions, two of the nine cases (Women's 57 kg and Men's + 80 kg) were classified as “AI errors”, while the remaining seven were deemed “controversial judgments”. In the “AI errors” category, the two cases involved determining whether a head strike was successful (Figure 7). A common factor was that the referees reviewed fewer camera angles, assuming the strikes were clear. Despite the Paris Olympic Taekwondo video review system supporting 360-degree playback, this limited view likely hindered the AI's ability to conduct a comprehensive multi-angle analysis, leading to incorrect judgments. In the “controversial judgments” category, all five panel members agreed that static images alone could not definitely resolve the seven cases. In two instances, on-site referees ruled head strikes as successful, but certain video angles suggested the defending athlete blocked the face with their hands (Figure 8). In the other five cases (i.e., where referees ruled unsuccessful head strikes, while AI deemed them successful), some angles clearly showed foot-to-helmet contact (Figure 9). While AI decisions aligned with its programmed criteria, a more accurate assessment would require referees to integrate additional factors, such as motion trajectory and a holistic video analysis, leveraging their experience and rule interpretation (29, 30). This suggests that AI, relying solely on frame-by-frame video analysis, may be limited in its ability to handle ambiguous scenarios requiring deeper contextual interpretation. Similar cases also frequently occurs in fields where AI is widely applied, such as medical and commercial sectors (e.g., AI's error rate increases when identify more complex clinical fracture cases, and it may be misled by patients' lies to make opposite diagnoses in psychological evaluations) (31–34). Analyzing such errors, Topol (2019) argues that Because AI models rely heavily on large datasets and pre-programmed decision algorithms during training, they consequently struggle with transfer learning in these unique scenarios, thereby hampering accurate diagnosis. Accordingly, this paper maintains that while AI can help reduce decision-making errors, proactive and interpretative judgments still require deep human involvement (35–37).

While AI demonstrates high consistency with international video review judges in Taekwondo competitions, it remains impractical to fully replace human referees in major events. AI still lacks the flexibility accurately interpret unique or complex situations (38). Additionally, the subjective decisions of human referees introduce unpredictability – an element that enhances spectator engagement and contributes to the sport's overall appeal. Many fans consider this unpredictability a key element of the charm of competitive sports (10, 36, 39). As Leslie (2024) notes, despite significant technological advancements, AI cannot fully replicate the role of human officials (40). Referees not only make nuanced judgments that go beyond AI's current capabilities, but also manage match tempo, enforce player discipline, and fulfill other critical functions. While spectators often criticize questionable calls, they remain unprepared to hand over full officiating authority to machines. In fact, occasional “human errors” are often seen as contributing to the drama and emotional intensity that define competitive sport (10, 36, 40). Moreover, the excessive objectivity and perceived algorithmic rigidity of AI judgments result in low acceptance of unfavorable decisions, rendering AI unsuitable for fully autonomous game adjudication (39).

Our findings demonstrates that AI cannot yet replace human referees in adjudication tasks. However, this study contends that AI can still play a significant role in Taekwondo officiating by assisting human referees in identifying critical evidence during video reviews, thereby enhancing both the accuracy and efficiency of decisions. For instance, head strike reviews often cause significant delays, as video review judges must repeatedly search through footage to pinpoint the moment of impact, especially since coaches have up to five seconds to request a review. Judges typically watch 5–10 s of footage to locate the strike and then review it from multiple angles. With AI assistance, cameras can track both athletes throughout the match. Once a review is requested, AI can quickly analyze the footage and provide key segments to referees, cutting review time by at least 80.9% - from 47 s (human) to just 9 s (AI). For simpler cases, such as out-of-bounds calls, AI can apply predefined algorithms to deliver an initial judgment, which judges can then confirm; or setting the model to trigger human review when the model confidence is lower than 90%, and to rely on AI for automatic judgment if it is higher than 90% (41). This approach could minimize unnecessary pauses, speed up the competition, and enhance its appeal to spectators.

While AI's role in major international Taekwondo competitions is currently limited to support, this is mainly because these events attract the world's best referees to ensure fairness at the highest level (42). However, in many municipal, provincial, and regional competitions, where resources for top-tier referees are often lacking, the value of AI becomes especially evident (43–45). In these cases, AI can directly assist in decision-making, enhance fairness, reduce event organizers' costs, and streamline event management. Additionally, AI can support the training of young local referees by assisting them identifying contentious rulings and retrieving adjudication results from relevant cases, enabling them to meet international judging standards. This, in turn, improves the overall quality of competitions (46) and advancing the global competitiveness of Taekwondo.

## 5 Conclusion

Our findings suggest that AI can serve as a valuable tool in Taekwondo video reviews, assisting referees by quickly identifying key penalty moments and improving overall efficiency. Preliminary research results show that the system can shorten the original review time by at least 81%, and can reduce operating costs and training costs such as labor expenses of referees in small and medium-sized competitions, thereby increasing the sustainability of Taekwondo events. Future research should enhance the AI's dynamic analysis capabilities by integrating multi-angle video fusion technology and motion trajectory prediction models to better handle minor contact and visual occlusion. While AI offers objectivity, the experience and adaptability of human referees remain essential. A hybrid model combining AI's preliminary judgment with referee review could strike an optimal balance between human expertise and computer assistance, enhancing both fairness and the flow of Taekwondo competitions. Finally, applying this AI-assisted framework to other sports (such as boxing, baseball, basketball and football etc.), could help assess its broader utility and generalizability.

## Data Availability

The original contributions presented in the study are included in the article/Supplementary Material, further inquiries can be directed to the corresponding author.
